# IL-6 Autoantibodies Predict Lower Platelet Counts and Altered Plasma Cytokine Profiles in Healthy Blood Donors: Results From the Danish Blood Donor Study

**DOI:** 10.3389/fmed.2022.914262

**Published:** 2022-06-24

**Authors:** Jakob Hjorth von Stemann, Ole Birger Vesterager Pedersen, Henrik Hjalgrim, Christian Erikstrup, Henrik Ullum, Joseph Dowsett, Lise Wegner Thørner, Margit Anita Hørup Larsen, Erik Sørensen, Morten Bagge Hansen, Sisse Rye Ostrowski

**Affiliations:** ^1^Department of Clinical Immunology, Rigshospitalet, Copenhagen University Hospital, Copenhagen, Denmark; ^2^Department of Clinical Immunology, Zealand University Hospital, Køge, Denmark; ^3^Department of Clinical Medicine, Faculty of Health and Medical Sciences, University of Copenhagen, Copenhagen, Denmark; ^4^Department of Epidemiology Research, Statens Serum Institut, Copenhagen, Denmark; ^5^Centre for Cancer Research, Danish Cancer Society, Copenhagen, Denmark; ^6^Department of Haematology, Rigshospitalet, Copenhagen University Hospital, Copenhagen, Denmark; ^7^Department of Clinical Immunology, Aarhus University Hospital, Aarhus, Denmark; ^8^Statens Serum Institute, Copenhagen, Denmark

**Keywords:** autoimmunity, IL-6, platelets, epidemiology, blood donors, cytokine autoantibodies, cytokines, thrombopoiesis

## Abstract

Cytokine-specific autoantibodies (c-aAb) represent a novel type of immune dysfunction. Though they have been detected in both patient cohorts and healthy individuals, and have immunomodulatory properties, the full extent of their influence remains unknown. Based on the critical role of several cytokines in thrombopoiesis, we investigated if there is an association between c-aAb and platelet variables in healthy individuals, with a specific focus on c-aAb against a known thrombopoietic cytokine, IL-6. Using platelet count and mean platelet volume in 3,569 healthy participants of the Danish Blood Donor Study as dependent variables, we performed a series of multivariate regression analyses using five cytokine autoantibodies, including IL-6 c-aAb, as independent variables. In men, high titers of IL-6 c-aAb were negatively associated with platelet counts (β = −24 ^*^10^9^/l (95% confidence interval −43 to −6), *p* = 0.008) and positively associated with mean platelet volume (β = 0.4 fL (95% confidence interval 0.0–0.7) *p* = 0.043). These associations were exacerbated when adjusting for undetectable C-reactive protein levels, which we used as a proxy for c-aAb mediated IL-6 inhibition *in vivo*. Furthermore, in a smaller subgroup, individuals with high vs. low titer IL-6 c-aAb had different profiles of plasma IL-6, IL-10, TNFα and TPO, further suggesting a functional inhibition of IL-6 by high titers of circulating IL-6 c-aAb. We therefore speculate that in addition to their immunomodulatory potential IL-6 c-aAb may interfere with thrombopoiesis – directly or indirectly – under normal physiological conditions. This study is the first to suggest an influence of c-aAb on platelets in healthy individuals, beyond their apparent effects on immune competence.

## Introduction

The immune system is a complicated network of interacting pathways and processes across multiple biologic systems and scales designed to combat pathogens (1). Furthermore, the immune system contributes to homeostasis throughout the body, including bone marrow (BM) function. Cytokines are immunological signaling molecules produced by cells of both the innate and adaptive immune system. Many cytokines are potent modulators of BM function where they govern many branches of hematopoiesis, either directly or indirectly through effects on other target organs such as the kidneys and the liver (2).

One such modulator is interleukin 6 (IL-6), a pleiotropic cytokine involved in both pro- and anti-inflammatory responses, ranging from promoting synthesis of acute-phase reactants such as C-reactive protein (CRP) to induction of the anti-inflammatory cytokine IL-10 (3, 4). Beyond its immunological properties, IL-6 also promotes thrombopoietin (TPO) release from liver cells, which in turn induces thrombopoiesis from megakaryocytes in the BM and promotes activation of mature platelets (5, 6). Furthermore, IL-6 has been confirmed *in vitro* to stimulate megakaryocytes directly through its associated receptor (7–9). Perpetually elevated IL-6 levels are associated with hypercoagulability (10), and both IL-6 and CRP levels have been used as markers for cardiovascular disease (11). In addition to IL-6-induced thrombopoiesis, a TPO independent acute thrombopoiesis pathway promoted by IL-1α was recently described (12), again involving a cytokine as a critical promoter of thrombopoiesis.

There is emerging evidence that naturally occurring cytokine-specific autoantibodies (c-aAb) represent a novel immunosuppressing phenomenon which is observed for a wide range of target cytokines in both healthy and diseased individuals (13–17), and may predict infection (18). Our group has developed and validated an in-house assay for the detection of c-aAb and reported high titers of c-aAb to be relatively common in Danish blood donors, including a 0.1–1% prevalence of high titers of IL-6 specific c-aAb (19–21). IL-6 c-aAb have been associated with bacterial infections and concurrent low CRP in several case studies (22–24). In hemostasis, c-aAb against IL-10, an IL-6 suppressor, are associated with an increased risk of major adverse cardiovascular events in kidney transplanted patients (25). Furthermore, treatment with antibodies directed against IL-6 [tocilizumab® (Actemra)] is associated with reductions in platelet counts (26). Our group has demonstrated that induced IL-6 c-aAb inhibits their target cytokine in murine models (27, 28), as well as *in vitro* verification of IL-6 inhibition by c-aAb isolated from healthy individuals (21, 29, 30). We have further discerned that IL-6 c-aAb in healthy donors are associated with undetectable CRP levels, and that the IL-6 inhibitory effect of c-aAb may be transferred via blood transfusion (20, 31). However, the understanding of wider biological influence of high-titer IL-6 c-aAb on phenotype in healthy individuals is still limited.

Based on the critical role of several cytokines in thrombopoiesis, the aim of the present study was to investigate the influence of naturally occurring c-aAb on platelet count and mean platelet volume (MPV) in healthy individuals, hypothesizing that high-titer c-aAb against IL-6 and IL-1α would be associated with reduced platelet count and ensuing increased MPV. To test the hypothesis, we investigated c-aAb against IL-1α, IL-6, IL-10, IFNα, and GM-CSF in 8,972 healthy participants of the Danish Blood Donor Study (DBDS), assessing the association between c-aAb titers and platelet measurements. Furthermore, in a small subgroup of selected individuals stratified according to high or low IL-6 c-aAb titers, we analyzed plasma levels of TPO and IL-6 associated cytokines (TNFα, IL-6 and IL-10) to reveal if high-titer IL-6 c-aAb functionally interfered with the cytokine network. To our knowledge this study is the first to investigate the physiological effect of c-aAb on thrombopoiesis and thus platelet count and volume.

## Materials and Methods

### Study Population

The present study is based on 8,972 participants screened for cytokine antibodies (c-aAb) against IL-1α, IL-6, IL-10, IFNα2, and GM-CSF, measured as Median Fluorescence Intensity (MFI). Participants were included in DBDS in 2010, and c-aAb were measured in plasma collected at the day of inclusion into the study using a custom made Luminex assay (19, 20). Platelet-related variables, including platelet count and MPV, were measured in EDTA-treated blood collected from the participants on a Sysmex XE-2100D using impedance. Sysmex measurements were initiated for the investigated blood donors in 2012. Participants were censored in the event of missing data, resulting in a final count of 3,569 participants in this study.

At time of inclusion study participants were healthy, completed a written health questionnaire (including declaration of Body Mass Index (BMI) and smoking habits), and were between 18 and 67 years old.

### Coding of Epidemiological Variables

BMI was calculated as self-reported weight (kg) / self-reported height (m)^2^ and was censored in case of anthropometric outliers (heights <1.5 m and >2.1 m, weight <45 kg and >160 kg). Smoking was coded as 1 if participants were smoking any number of cigarettes at inclusion date. Age, usage of oral contraceptives (OC) and comorbidity were also defined at inclusion date. We calculated Charlson comorbidity according to 15 years of disease history for the participants, as previously described, according to data from the Danish National Patient Registry (32). Donation history was defined as number of whole-blood donations within 1 year prior to c-aAb measurement. A separate 1-year donation history was generated relative to the date of platelet measurements. Data on prescription of antimicrobials were obtained from the Danish National Prescription Registry, which records all pharmacy prescriptions of drugs in Denmark, from 1994 and onwards (33). Participant prescription history of antimicrobials in general (ATC = J01) were defined as having had any prescriptions of the investigated antimicrobials within 1 year prior to c-aAb measurement. As done with donation histories above, a separate 1-year prescription history was generated relative to the date of platelet measurements. CRP concentration was transformed into a binary variable classified as “undetectable” (<0.1 mg/L) or “detectable” (≥0.1 mg/L).

### Coding of Cytokine Autoantibody Independent Variables

Participants were stratified into low, intermediate, and high titers of c-aAb as the independent variables. As in previous publications (20), we defined high titers of c-aAb as Median Fluorescence Intensity (MFI) signals above the 99th percentile in the population, low c-aAb titers as MFI values equivalent to or below the average negative control signal + 4 standard deviations (SD), and intermediate titers were defined as signals between these values. Participants with high titers of c-aAb were considered the most likely to experience cytokine inhibition, and thus altered thrombopoiesis. Based on our previous finding of very different c-aAb titers associated with sex (20), separate c-aAb titers were defined for men and women.

### Cytokine Screening

#### Sample Selection

From the main cohort, we collected additional plasma samples from a subgroup of 69 individuals with either high vs. low IL-6 c-aAb titer, for further cytokine analysis. Thus, 36 of these had signals for the five originally screened c-aAb below the previously defined low-titer threshold, and were thus considered IL-6 c-aAb low-titer, whereas 33 individuals were among the 99th percentile of IL-6 c-aAb signals in the original DBDS cohort, and thus considered IL-6 c-aAb high-titer. All the samples were from individuals who had undetectable levels of CRP. Of the collected samples 50% of the c-aAb low-titer samples, and 52% of the c-aAb high-titer samples were men. As the samples originally used for DBDS cohort inclusion were unavailable, samples from the closest possible blood donation time-point were chosen.

#### Cytokine Measurements

The concentration of IL-6, IL-10 and TNFα were measured in all collected plasma samples in pg/ml using the Magnetic Luminex® Performance kit from R&D systems (Catalog number LUHM000) and analyzed on our Luminex 200 instrument. The cytokine panel was chosen in terms of viable indicators of the IL-6 signaling axis as well as thrombopoiesis. C-aAb against IL-1α, IL-6, IL-10, IFNα2, and GM-CSF were assessed using the aforementioned custom-made assay (19).

### Statistical Analyses

To investigate the association between potentially cytokine-inhibiting c-aAb titers and platelet count/MPV in healthy blood donors, we applied multiple linear and logistic regression models using platelet count and MPV as dependent variables. Analyses were all stratified by sex and performed using STATA (STATA/MP15 for PC, StataCorp, College Station, TX). *p-*values below 0.050 were considered statistically significant.

Linear regression models used continuous platelet count and MPV measurements as dependent variables, whereas new variables for high (above 90th percentile platelet count/MPV value) or low (below 10th percentile platelet count/MPV value) levels of platelet variable were generated for the logistic models. As there was no overlap between samples with c-aAb and platelet measurements, measured c-aAb titers were compared to the earliest available platelet measurements relative to c-aAb sample date for the respective participant. Participants were censored in the event of a time span of more than 2 years between c-aAb and platelet measurements.

#### Selection of Covariates for Multivariate Regressions

All multivariate analyses were adjusted for age, smoking, BMI, OC, comorbidity, 1-year donation history, and 1-year history of antimicrobial prescriptions. All selected covariates were associated with either platelet or c-aAb measurements in preceding univariate tests with *p*-values of <0.050. Univariate tests consisted of students *t*-tests to test for associations between continuous vs. binary variables (such as platelet measurements vs. smoking), and chi-squared tests for comparison of high c-aAb titers to binary variables. Pearson's correlations were used to compare platelet measurements with other continuous variables such as age or BMI. In the event of missing data for an included dependent or independent variable, participants were censored from analysis. As the results for analyses using donation/prescription histories defined relative to the date of c-aAb measurement were highly similar to those using histories defined relative to the date of platelet measurement, we chose to present results from the former in this manuscript.

#### Analysis of Multiple C-aAb as Predictors of Platelet Variables

In a subsequent series of linear regressions, we analyzed the combined presence of c-aAb as predictors of the closest platelet measurements, stratified according to overlap between combinations of low, intermediary, and high titers of c-aAb. All possible c-aAb combinations were tested two at a time (e.g., IL-1α and IL-6 c-aAb, IL-1α and IL-10 c-aAb etc.) and adjusted as described above. Participants with low titers of both investigated c-aAb were used as reference.

#### C-aAb and CRP as Predictors of First Measurement of Platelet Variables

Further analyses incorporated CRP measurements into the multivariate analyses. CRP has previously been observed to be negatively associated with IL-6 c-aAb titers and is suggested as an indicator of cytokine suppression. We therefore reasoned that individuals presenting both low CRP concentration and high c-aAb titers represented the most likely cases of cytokine suppression and investigated the cumulative effect of IL-6 c-aAb titers and CRP as predictors of platelet measurements. Individuals with low IL-6 c-aAb and detectable CRP were used as baseline since these were inferred as a phenotype in one end of the investigated spectrum (no c-aAb and hence no influence on CRP).

#### C-aAb and CRP as Predictors of Mean/Median Platelet Variables

In supplementary analyses, dependent variables were defined as an individuals' mean/median platelet count and MPV, corresponding to platelet measurements taken within 5 years of c-aAb measurement. Linear regressions were performed with or without CRP stratification and with the same independent variables as above.

#### Subgroup Analysis of C-aAb, TPO and IL-6 Associated Cytokines

Except for TPO the measured cytokines and c-aAb all followed non-normal distributions. Associations between IL-6 concentrations and IL-10 and TNFα, as well as associations between c-aAb MFI and cytokine concentrations, were assessed by Spearman correlation. Association to TPO concentration were assessed through linear regression, with TPO as the dependent variable. Subsequent analyses stratified the above association studies according to the IL-6 c-aAb titer classification of the samples.

Having IL-6 concentrations above the 75th percentile was defined as having high concentrations of the cytokine. The presence of cytokine-high or non-high individuals were compared to measured c-aAb titers through Wilcoxon rank sum test, and to the IL-6 c-aAb high-titer/ low-titer groups through chi squared test. The subgroup analyses were not stratified by sex due to the limited sample size.

### Ethics Approval and Consent to Participate

Oral and written informed consent has been obtained for all participants at the time of inclusion into DBDS. The study has been approved by both the Danish health research ethics committee system (CVK-1700407 and SJ740) and the Danish Data Protection Agency (P-2019-99).

## Results

### Characteristics of the Cohort

Our initial sample population comprised 8,972 DBDS participants, with previously measured titers of cytokine-specific autoantibodies (c-aAb) against IL-1α, IL-6, IL-10, IFNα, and GM-CSF (20). A demographic overview of the participants with complete data for all variables relevant to this study, including platelet measurements (*n* = 3,569) is shown in Table 1. All displayed variables (Age, smoking, BMI, OC, prescription history, donation history, comorbidity and CRP) were associated with c-aAb, platelet count and/or MPV in univariate tests. The population comprised 46% women and 54% men. Men were older than women, donated more frequently prior to c-aAb measurement, had more often CRP levels below the detection limit and had higher IL-1α c-aAb and IL-10 c-aAb, whereas women had higher levels of platelet count, IL-6 c-aAb, GM-CSF c-aAb, higher BMI, and filled more antimicrobial prescriptions within 1-year follow-up as compared with men (Table 1).

**Table 1:** Characteristics of the study population.

	**Women**	**Men**	***p*-value^**a**^**
Number of participants (%)	1,646 (46)	1,923 (54)	
Age (years)^b^	40 (±12)	41 (±12)	0.016
Smoking (current) %^c^	15.9	16.6	0.588
BMI^b^	26(±4)	25 (±3)	<0.0001
Use of Combined oral contraceptives (%)^c^	27		
Platelet count (10^−9^/L)^b^	250 (±55)	223 (±44)	<0.0001
MPV (fL)^b^	11.6 (± 0.9)	11.6 (±0.9)	0.908
Filled at least one prescription for antimicrobial (%)^d^	31	17	<0.0001
Donation history^b^	1.74 (± 1.05)	1.97 (± 1.00)	<0.0001
Comorbidity score>1 (%)^c^	2.79	3.27	0.559
CRP (mg/L)^b^ Undetectable: < 0.1 mg/L (%)^c^	1.61 (± 2.28) 17.2	1.03 (± 1.61) 21.9	<0.0001 <0.0001
IL-1α c-aAb signal (MFI)^b^	542 (± 1,429)	791 (± 2,098)	<0.0001
IL-6 c-aAb signal (MFI)^b^	651 (± 1,475)	631(± 1,829)	0.035
IL-10 c-aAb signal (MFI)^b^	131 (± 458)	139 (± 373)	<0.0001
IFNα c-aAb signal (MFI)^b^	159 (± 525)	162 (± 452)	0.149
GM-CSF c-aAb signal (MFI)^b^	299 (± 1,372)	224 (± 877)	0.050

a *Students t-test were used for comparison of continuous variables across sex (age, BMI, platelet count, MPV, log-transformed CRP, donation history, c-aAb levels), and chi squared tests were used for comparison of categorical and ordinal variables (smoking, OC use, prescription history, comorbidity, undetectable/detectable CRP).*

b *Data presented as mean ± SD.*

c *Data presented as percentage of population.*

d *ata presented as incidence proportion, within 1 year of follow-up*.

### Association Between C-aAb, Platelet Count, and MPV

Using univariate and multivariate linear regression models, IL-6 c-aAb titers displayed a negative independent association with platelet count for men, for both intermediary titers (independent association, β = −7, (95% CI −11 to −3), *p* = 0.001) and high titers of IL-6 c-aAb (independent association, β = −24 (−43 to −6), *p* = 0.008) (Table 2A). Furthermore, in men we observed an independent positive association between IL-6 c-aAb titers and MPV in the adjusted regressions both for intermediary (β = 0.1 (0.0–0.2) *p* = 0.046) and high titers of IL-6 c-aAb (β = 0.5 (0.0–0.7) *p* = 0.043).

**Table 2A:** C-aAb as predictors of platelet count and mean platelet volume in men.

**C-aAb specificity**	**Platelet measurement**	**C-aAb titer**	***n* ^a^**	**Univariate regression coefficient (95%CI)**	**Multivariate regression coefficient (95%CI)^**b**^**	***p*-value^**c**^**
IL-6	Platelet count	Intermediary^d^	1,922	−5 (−8 to −2)	−7 (−11 to −3)	0.001
		High^e^		−13 (−28 to −2)	−24 (−43 to −6)	0.008
	MPV	Intermediary^d^	1,920	0.1 (0.0–0.1)	0.1 (0.0–0.2)	0.046
		High^e^		0.3 (−0.0–0.6)	0.5 (0.0–0.7)	0.043
IL-1α	Platelet count	Intermediary^d^	1,922	0 (−3–3)	−2 (−6–2)	0.375
		High^e^		−7 (−22–8)	−9 (−2–11)	0.422
	MPV	Intermediary^d^	1,920	0.0 (−0.1–0.0)	−0.1 (−0.1–0.0)	0.181
		High^e^		−0.1 (-0.3–0.3)	0.1 (−0.4–0.5)	0.793
IL-10	Platelet count	Intermediary^d^	1,922	−2 (−5–1)	−2 (−5 −2)	0.368
		High^e^		−6 (−21–8)	−12 (−29–8)	0.262
	MPV	Intermediary^d^	1,920	0.0 (−0.1– 0.1)	0.0 (−0.1–0.1)	0.477
		High^e^		0.1 (−0.3– 0.4)	0.1 (−0.4–0.6)	0.605
IFNα	Platelet count	Intermediary^d^	1,922	−1 (-4–2)	1 (−4–5)	0.951
		High^e^		0 (−14– 14)	1 (−20–23)	0.988
	MPV	Intermediary^d^	1,920	0.1 (−0.1– 0.1)	0.0 (−0.1–0.1)	0.387
		High^e^		0.0 (−0.3 −0.3)	0.0 (−0.4–0.5)	0.898
GM-CSF	Platelet count	Intermediary^d^	1,922	−2 (−6– 3)	1 (−5–7)	0.684
		High^e^		0 (−14– 15)	−2 (−26–21)	0.774
	MPV	Intermediary^d^	1,920	0.0 (−0.1– 0.1)	−0.1 (−0.2– 0.1)	0.078
		High^e^		0.1 (−0.2−0.3)	0.0 (-0.5– 0.5)	0.992

a *Number of participants in adjusted linear regressions.*

b *Adjusted for age, smoking, BMI, 1-year donation history, 1-year history of antimicrobial prescriptions, comorbidity, and distance between c-aAb and closest platelet measurement. The first platelet count and MPV within 2 years of c-aAb measurement was used as the dependent variable.*

c *p values shown correspond to the adjusted regressions.*

d *Intermediary c-aAb titer defined as MFI values above negative control +4 SD, but below the 99th percentile of MFI. Compared to c-aAb low individuals, with MFI< negative control +4 SD.*

e *High c-aAb titers defined as MFI values above the 99th percentile of MFI. Compared to c-aAb low individuals, with MFI < negative control +4 SD*.

In supplementary multivariate regression analyses, we also found IL-6 c-aAb in men to be negatively associated with median platelet counts calculated over a five-year period. Associations were significant for both intermediary (β = −5 (−9 to −2) *p* = 0.002) and high titers of IL-6 c-aAb (β = −19 (−34– −3) *p* = 0.020). In men, similar results were found for mean platelet counts. No associations between IL-6 c-aAb and mean or median MPV across 5 years were observed. In multivariate logistic regressions, men with high-titer IL-6 c-aAb had increased odds for low platelet count (below the 10th percentile), as well as increased odds for high MPV (above the 90th percentile, Supplementary Table S1).

No other c-aAb were associated with platelet count or MPV (Table 2B), and no significant associations were observed in women. Furthermore, no cumulative effect of c-aAb was observed when investigating individuals with intermediary or high titers of two c-aAb simultaneously as predictors of platelet variables (data not shown).

**Table 2B:** C-aAb as predictors of platelet count and mean platelet volume in women.

**C-aAb specificity**	**Platelet measurement**	**C-aAb titer**	***n* ^a^**	**Univariate regression coefficient (95%CI)**	**Multivariate regression coefficient (95%CI)^**b**^**	***p*-value^**c**^**
IL-6	Platelet count	Intermediary^d^	1,643	1 (−3–5)	5 (−1–10)	0.122
		High^e^		9 (−9–27)	9 (−14–37)	0.366
	MPV	Intermediary^d^	1,640	0.0 (−0.1–0.1)	0.0 (−0.1–0.1)	0.840
		High^e^		0.0 (−0.3–0.3)	0.1 (−0.3–0.5)	0.776
IL-1α	Platelet count	Intermediary^d^	1,643	0 (−4–5)	1 (−4–6)	0.764
		High^e^		−8 (−27–12)	−1 (−28–21)	0.755
	MPV	Intermediary^d^	1,640	0.0 (−0.1–0.1)	0.0 (−0.1–0.1)	0.568
		High^e^		0.2 (−0.2–0.5)	0.3 (−0.1–0.7)	0.120
IL-10	Platelet count	Intermediary^d^	1,643	1 (−3–4)	2 (−3–7)	0.479
		High^e^		11 (−8–30)	18 (-6–42)	0.150
	MPV	Intermediary^d^	1,640	0.0 (−0.1–0.1)	−0.1 (−0.1–0.1)	0.264
		High^e^		−0.3 (−0.7–0.1)	−0.3 (−0.7–0.2)	0.298
IFNα	Platelet count	Intermediary^d^	1,643	0 (−4–3)	−1 (−6–4)	0.612
		High^e^		5 (−15–25)	4 (−27–37)	0.687
	MPV	Intermediary^d^	1,640	0.0 (−0.1–0.1)	0.0 (−0.1–0.1)	0.750
		High^e^		−0.1 (−0.4–0.2)	0.0 (−0.5–0.5)	0.925
GM-CSF	Platelet count	Intermediary^d^	1,643	−3 (−9–3)	−1 (−9–8)	0.895
		High^e^		−4 (−22–14)	−6 (−32–19)	0.625
	MPV	Intermediary^d^	1,640	0.0 (−0.1–0.1)	−0.1 (−0.2–0.1)	0.492
		High^e^		0.1 (−0.2–0.4)	−0.1 (0.5–0.3)	0.697

a *Number of participants in adjusted linear regressions.*

b *Adjusted for age, smoking, BMI, 1-year donation history, 1-year history of antimicrobial prescriptions, comorbidity, oral contraceptives and distance between c-aAb and closest platelet measurement. The first platelet count and MPV within 2 years of c-aAb measurement was used as the dependent variable.*

c *p values shown correspond to the adjusted regressions.*

d *Intermediary c-aAb titer defined as MFI values above negative control +4 SD, but below the 99th percentile of MFI. Compared to c-aAb low individuals, with MFI< negative control +4 SD.*

e *High c-aAb titers defined as MFI values above the 99th percentile of MFI. Compared to c-aAb low individuals, with MFI< negative control +4 SD*.

### Association Between Undetectable CRP and High-Titer IL-6-Specific C-aAb With Platelet Count and MPV

**Figure 1:**
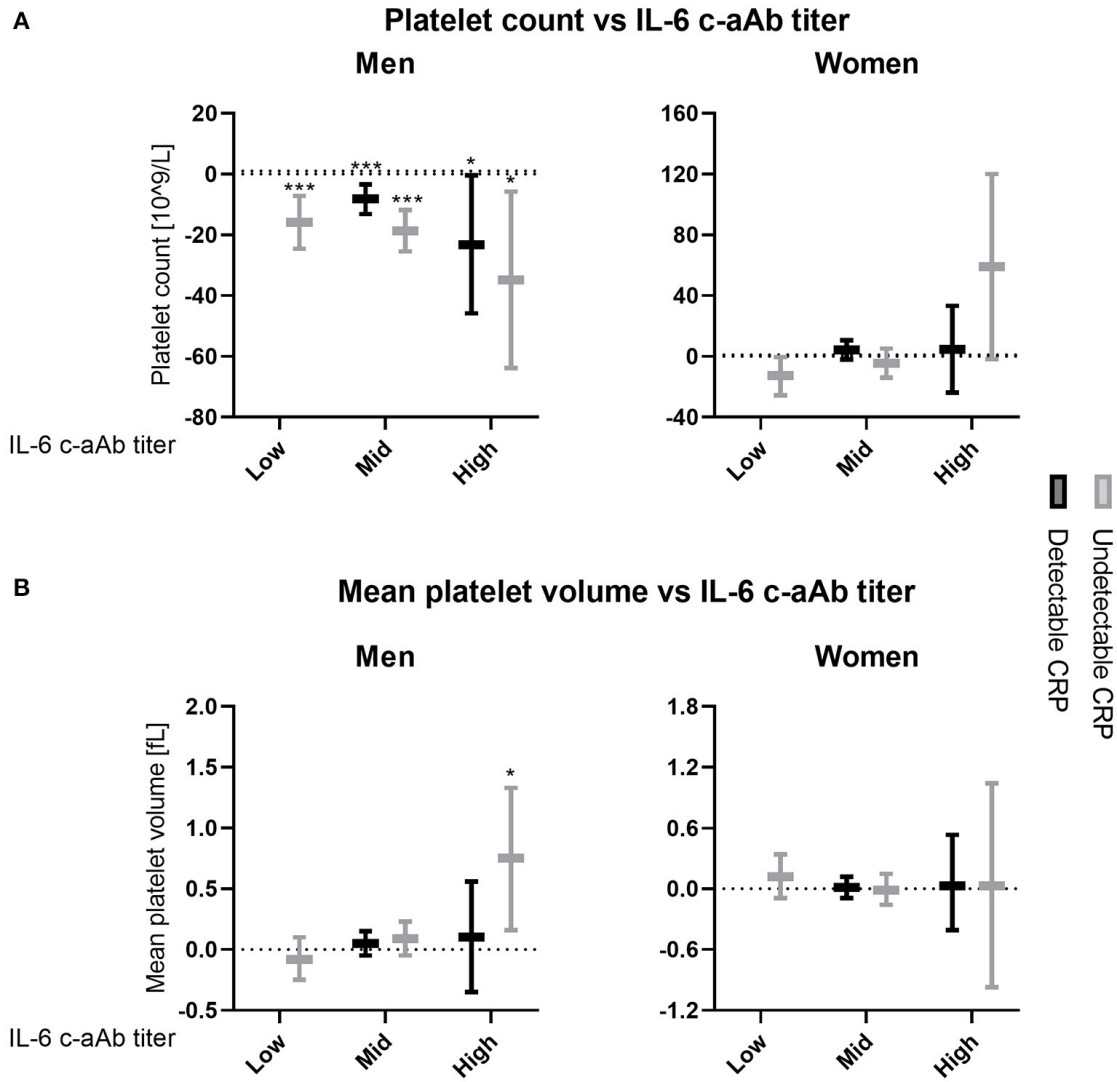
Association between IL-6 c-aAb and CRP levels with platelet count and MPV. Multivariate linear regression models were used to investigate levels of IL-6 c-aAb and CRP as predictors of **(A)** platelet count and **(B)** MPV. Analyses were performed for all combinations of c-aAb and CRP levels as independent variables, with individuals with low titer c-aAb and detectable CRP used as the baseline. High titers of c-aAb were defined as MFI above the 99th percentile, intermediary c-aAb titers were defined as MFI values > negative control + 4SD and < the 99th percentile, and low titers of c-aAb were defined as below the intermediate titers. Separate c-aAb titers were calculated for women and men. Undetectable CRP was defined as having CRP < 0.1 mg/L, and detectable CRP as having CRP ≥0.1 mg/L. Other independent variables include titers of c-aAb, age, smoking, BMI, oral contraceptives, 1-year prescription history of antimicrobials, 1-year donation history and comorbidity. For binary variables usage of contraceptives and having had at least one prescription a year before c-aAb measurement were coded as 1. Data are presented as regression coefficients with 95% Confidence Interval. * denotes a *p* value of ≤ 0.05, ***p ≤ 0.001.

Using multivariate linear regression models stratified by CRP in men, a dose-dependent negative association between IL-6 c-aAb titers and platelet count was observed for both individuals with undetectable and individuals with detectable levels of CRP, using individuals with low IL-6 c-aAb titers and detectable CRP levels as a reference group (Figure 1).

In men with detectable CRP, as a proxy for negligible IL-6 c-aAb *in vivo* inhibition of this axis, negative associations with platelet counts were observed for both intermediary and high-titer IL-6 c-aAb, relative to individuals with low titer IL-6 c-aAb and detectable CRP (β = −8 (−13 to −3) *p* = 0.001 and β = −23 (−46 to −0.5) *p* = 0.045, respectively, Figure 1A).

For men with undetectable levels of CRP, as a proxy for some IL-6 c-aAb *in vivo* inhibition of this axis, the observed negative associations of IL-6 c-aAb with platelet count featured larger effect sizes, and stronger statistical significance for both intermediary and high-titer IL-6 c-aAb (β = −19 (−26 to −12) *p* < 0.001 and β = −35 (−64 to −6) *p* = 0.019, respectively) in comparison to men with detectable CRP. In men with undetectable CRP and high-titer IL-6 c-aAb, a positive association was also observed between IL-6 c-aAb and MPV (β = 0.8 (0.2–1.3) *p* = 0.012).

**Figure 2:**
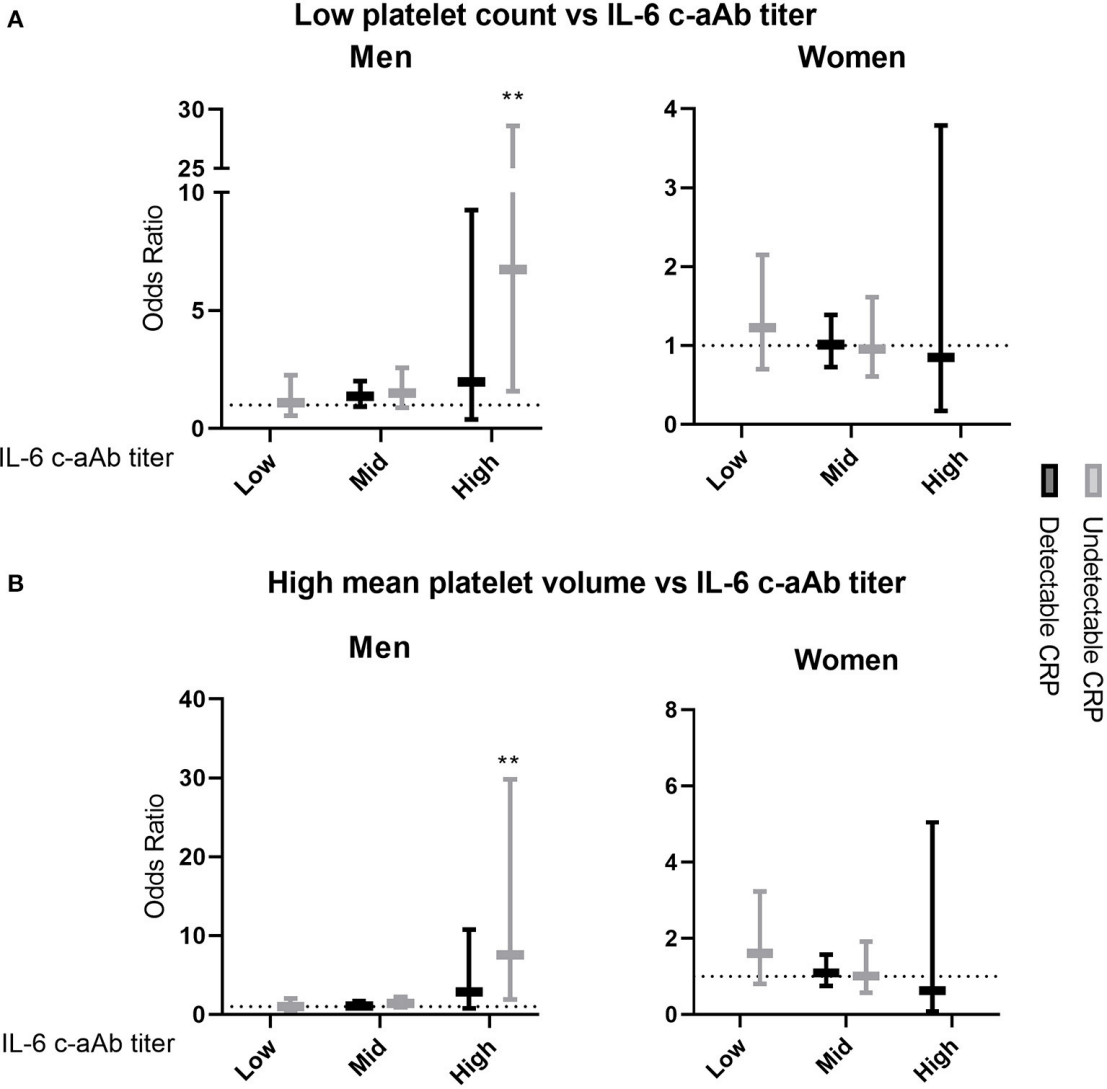
Association of IL-6 c-aAb and CRP levels with having low or high platelet variables. Multivariate logistic regression models were used to investigate levels of IL-6 c-aAb and CRP as predictors of the odds of having **(A)** low platelet count and **(B)** high MPV. Analyses were performed for all combinations of c-aAb and CRP levels as independent variables, with individuals with low titer c-aAb and detectable CRP used as the baseline. Low or high levels of platelet count and MPV were defined as scores below or above the 10th and 90th percentiles, respectively. High titers of c-aAb were defined as MFI above the 99th percentile, intermediary c-aAb titers were defined as MFI values > negative control + 4SD and < the 99th percentile, and low titers of c-aAb were defined as below the intermediate titer. Separate c-aAb titers were calculated for women and men. Undetectable CRP was defined as having CRP <0.1 mg/L, and detectable CRP as having CRP ≥0.1 mg/L. Other independent variables include titer of c-aAb, age, smoking, BMI, oral contraceptives, 1-year prescription history of antimicrobials, 1-year donation history and comorbidity. For binary variables usage of contraceptives and having had at least one prescription per year before c-aAb measurement were coded as 1. Data are presented as OR with 95% Confidence Interval (CI). ** denotes a *p*-value of ≤ 0.01.

In multivariate logistic regressions, men with high-titer IL-6 c-aAb and undetectable CRP, had increased odds for low platelet count (OR = 6.7 (1.6–28.6), *p* = 0.010) as well as increased odds for high MPV (= 7.6 (1.9–29.9), *p* = 0.004, Figure 2).

No significant associations were observed between IL-6 c-aAb, CRP and either platelet variable in women in both linear and logistic regression analyses.

### Association of High-Titer IL-6 C-aAb With Altered Cytokine Levels in Select Cases

In a subgroup study of samples from later time-points from selected IL-6 c-aAb high-titer and IL-6 c-aAb low-titer individuals from the cohort, we observed c-aAb titers to be consistent with those seen in the original samples (data not shown). Analyzing all high and low IL-6 c-aAb titer individuals together, we found IL-6 plasma concentration to be positively correlated with IL-10 and TNFα concentration (Spearman correlations ρ = 0.65 *p* < 0.0001 and ρ = 0.45 *p* < 0.001, respectively) but not with TPO (Table 3). Furthermore, IL-6 c-aAb at the time of cytokine measurement were positively correlated with IL-6 plasma concentrations, both for all samples (ρ = 0.38 *p* = 0.001) and especially for samples with the highest IL-6 c-aAb titers (ρ = 0.66 *p* < 0.0001). Furthermore, Individuals with the highest IL-6 cytokine concentrations (above the 75th percentile) were predominantly, and significantly in the IL-6 c-aAb high-titer group (chi squared test, *p* = 0.035). IL-6 c-aAb in general as well as the classification as being IL-6 c-aAb high or low-titer, were not associated with concentrations of IL-10, TNFα or TPO.

**Table 3:** Associations of plasma IL-6 with other cytokines.

	**IL-10^**a**^**	**TNFα^a^**	**TPO^**b**^**
All samples	ρ = 0.65, *p* < 0.0001	ρ = 0.45, *p* < 0.0001	ρ = –*7, p = 0.066*
C-aAb low-titer group	ρ = 0.88, *p* < 0.0001	ρ = 0.64, *p* < 0.0001	ρ = −7, *p* = 0.615
IL-6 c-aAb high-titer group	ρ = 0.33, *p* = 0.105	ρ = 0.22, *p*= 0.286	ρ = −7, *p* = 0.029

a *Spearmans Rho and p-values for associations between continuous non-parametric plasma cytokine concentrations (pg/mL), either for all samples or stratified by c-aAb titer group.*

b *Linear regression coefficients and p-values for associations between continuous cytokine concentrations, either for all samples or stratified by c-aAb titer group, due to normal distribution of TPO concentration in the samples*.

When grouping the analyses based on IL-6 c-aAb low or high titers, we observed that IL-6 cytokine concentrations only correlated positively with IL-10 and TNF-α in the c-aAb low-titer group. A negative correlation was seen between IL-6 and TPO concentration in the IL-6 c-aAb high-titer group, but not in the c-aAb low-titer group (Table 3).

Finally, we investigated samples with high vs. low levels of IL-6 separately, due to the notable overlap between high levels of IL-6 concentration and high titers of IL-6 c-aAb because we wanted to see if the previously observed cytokine interactions were still present for this group, in spite of the present high titer IL-6 c-aAb. In samples with low IL-6 levels (below 75th percentile), we observed positive correlations between IL-6 and IL-10 and between IL-6 and TNFα (ρ = 0.84, *p* < 0.001 and ρ = 0.74, *p* < 0.001, respectively). In contrast, in samples with high IL-6 levels (above 75th percentile) we observed either no significant correlations, or a negative correlation between IL-6 and IL-10, suggesting that the interplay observed in the individuals with low IL-6 cytokine were not present.

## Discussion

Natural cytokine-specific autoantibodies show evidence of immunomodulatory effects, even in healthy individuals. To further elucidate the phenomenon, this study investigated the potential impact of c-aAb on platelet count and MPV in healthy individuals, due to the involvement of cytokines, such as IL-6, in thrombopoiesis. In men we found statistically significant associations between high titers of IL-6 specific c-aAb and platelet count and MPV, respectively. None of the other four investigated c-aAb displayed significant associations to platelet variables. The negative association was stronger for high compared to intermediate IL-6 c-aAb titers. Furthermore, in a smaller sub-study we observed altered concentrations and cytokine inter-correlations in IL-6 c-aAb high-titer individuals compared to c-aAb low-titer individuals. Based on the established inhibitory potential of high-titer c-aAb on their target cytokine (17, 21, 27, 28, 34), we speculate that the negative association between IL-6 c-aAb and PLT may be explained by a direct or indirect IL-6-induced influence on thrombocytopoiesis in individuals with high titers of IL-6 c-aAb, resulting in a lower baseline platelet count and thus higher MPV.

We introduced CRP levels as a factor into our cohort analysis to bolster the categorization of IL-6 c-aAb with inhibitory potential. CRP is another prominent downstream target of IL-6, and high titers of IL-6 c-aAb have previously been associated with low or undetectable CRP levels (22–24). We have previously confirmed the association between high titers of IL-6 c-aAb and undetectable CRP in the present population of healthy blood donors, interpreting it as an indirect indicator of c-aAb mediated inhibitory potential (20). Therefore, we reasoned that individuals expressing both undetectable CRP and elevated titers of IL-6 c-aAb, would be the most likely to have affected thrombopoiesis through inhibition of IL-6, because IL-6 induces both CRP and TPO in the liver. Indeed, the association between IL-6 c-aAb and platelet variables was exacerbated when individuals were further stratified by CRP-levels; individuals with both IL-6 c-aAb and undetectable CRP expressed greater effects sizes on platelet variables than individuals with only elevated titers of IL-6 c-aAb. The same relationship was observed when investigating IL-6 c-aAb as predictors of having the lowest 10 percentile platelet count, as more positive odds for low platelet count were observed for men with undetectable CRP and the highest titers of IL-6 c-aAb. The lack of statistically significant associations between platelet variables and other c-aAb further suggests that the observed potential inhibition of thrombopoiesis is specific to c-aAb targeting the IL-6 pathway.

The positive association between IL-6 c-aAb and MPV was observed in men with high titers of c-aAb and undetectable levels of CRP predicting the largest effect sizes. It is well-established that lower platelet count is associated with increased MPV (35) and we infer that this is the case for the observed associations. The observed increased MPV in individuals with elevated IL-6 c-aAb may thus reflect a physiological compensation in response to slight IL-6 c-aAb induced suppression of platelet counts. Alternately, it is possible that IL-6 c-aAb-mediated immunomodulation influences platelet turnover rather than platelet synthesis with increased loss of smaller platelets.

The sex-specificity of the c-aAb effects is intriguing. It is well-established that sex influences both the innate and adaptive immune system, with women generally being generally better antibody producers than men, and with recognized higher antibody titers as well (36). In the present study we observed higher IL-6 and GM-CSF c-aAb titers in women compared with men. Furthermore, some sex differences in IL-6 physiology are known, including elevated levels of IL-6 in pregnant/postmenopausal women and differing IL-6 responses associated with trauma (37–39). Thus, it is possible that men may express lower tolerance for IL-6-specific c-aAb. Furthermore, there are established differences between platelet count for men and women with women having higher platelet count across most ages (40, 41). This is in accordance with findings in the present study; however, this study cannot establish the biological causes of the observed difference between genders which will be a point of interest in future studies.

In order to further elucidate whether the high titers of IL-6 c-aAb observed in the cohort could induce functional inhibition of the IL-6 cytokine and hence the TPO axis, we performed a small scale cytokine screening on select cases with low and high IL-6 c-aAb titers selected from the cohort. In this subgroup IL-6 cytokine was found to be positively correlated to IL-10 and TNFα but had no correlation to TPO. To our knowledge, no correlation has been published between plasma levels of IL-6 and TPO in healthy individuals. We also found a positive correlation between IL-6 cytokine concentrations and IL-6 c-aAb titer. This correlation was carried by the IL-6 c-aAb high-titer group which contained all the highest observed IL-6 concentrations. The true concentration of plasma IL-6 is hard to determine in the context of IL-6 c-aAb, as the available measurable cytokine depends on the binding specificity and activity of the present c-aAb in relation to the antibodies of the assay (42). However, the fact that the concentration of detectable IL-6 cytokine was *higher* than in individuals with no potentially interfering IL-6 c-aAb indicates that the positive association is valid and could indicate that the cytokine is stored in antibody complexes in high-titer individuals. This is a phenomenon we have previously described in healthy individuals (21). However, beyond concentration *per se*, the key factor is the bioactivity of the IL-6 cytokine, which we sought to investigate through the levels of IL-6-associated cytokines IL-10, TNFα, and TPO. We found that in IL-6 c-aAb high-titer individuals the positive correlations otherwise seen between IL-6 cytokine and IL-10 and TNFα observed in the low-titer group were non-existent. Despite IL-6 being an inducer of TPO production, a negative association between IL-6 and TPO was found, exclusively in the IL-6 c-aAb high-titer group.

Taken together, these results suggest that the presence of high-titer IL-6 c-aAb could disrupt the normal interplay between IL-6 and the investigated cytokines including downstream TPO. However, it is important to note that IL-6 may affect thrombopoiesis directly, and independently of TPO through the generation of megakaryoblasts from CD34 cells or stimuli of megakaryoblasts themselves (7–9, 43). Furthermore, morphologically normal platelets can still be produced in TPO knockout mice, suggesting that TPO is sufficient but not necessary for platelet production (44).

Combined with the cohort data as well as prior studies (20), it thus seems possible that IL-6 c-aAb may influence BM function indirectly via modulation of TPO release from the liver or directly through interaction with megakaryocytes and their predecessors in order to slightly cap the level of measurable platelets.

The observed possible inhibitory effect of IL-6 c-aAb on platelets raises the question of whether other c-aAb may influence other pathways of thrombopoiesis, such as IL-1α mediated acute thrombopoiesis. Though we did not observe an association between IL-1α specific c-aAb and platelet variables in this study, it is worth noting that this study concerned healthy individuals and featured a time lapse between c-aAb and platelet measurement which might have masked any such effects. We speculate that measuring IL-1α c-aAb in actively hemorrhaging and/or thrombocytopenic patients such as trauma or septic patients could be highly interesting specifically for older men who have an increased likelihood of high-titer IL-1α c-aAb (45).

To our knowledge, this is the first study that suggests a relationship between c-aAb and platelets, though it must be emphasized that our results only supports an association and not a causal relationship. A limitation of the study is the time gap between biomarker measurements which means that c-aAb titers at the time of platelet measurement are not known, and vice-versa. However, we know that elevated c-aAb titers may remain stable for years (21, 34, 46), which lets us consider the present findings trustworthy, even more so in light of IL-6 c-aAb associating negatively with not just the first platelet count after c-aAb measurement, but the overall mean/median platelet counts of individuals over a period of 5 years. Due to the nature of this investigation as a cross-sectional population study of healthy individuals and the routine measurements of platelets we believe risk of any systemic biases in this study are low. Moreover, the nature of the population as healthy blood donors also means the absence of any truly thrombocytopenic individuals from this study. Ultimately the study is exploratory in nature, and contains risk for type 1 errors due to the multiple analyses performed. The cytokine sub-study is limited by the sample size and the lack of perfect overlap between CRP, platelet, and cytokine measurements. Furthermore, the IL-6 – TPO-platelet axis is speculated to be triggered by inflammation (5), indicating that our study population of healthy individuals as well as cases with low CRP may underestimate the effect of IL-6 c-aAb on the axis. Further studies investigating IL-6 c-aAb and platelet counts at the point of infections are warranted.

In conclusion, this study is the first to describe that high-titer IL-6 c-aAb may influence thrombopoiesis – directly or indirectly – under normal physiological conditions, thus suggesting novel effects of c-aAb on the bone marrow function that goes beyond their previously described effects on immune competence. This makes c-aAb prime targets for further investigations in immunocompromised or otherwise vulnerable patients.

## Data Availability Statement

The datasets presented in this article are not readily available because with reference to Danish law, we can not upload data, but data may be made available by contacting the author. The access to data still has to be in compliance with Danish law. Requests to access the datasets should be directed to jakob.hjorth.von.stemann@regionh.dk.

## Ethics Statement

The studies involving human participants were reviewed and approved by the Danish Health Research Ethics Committee System (CVK-1700407 and SJ740) and the Danish Data Protection Agency (P-2019-99). The patients/participants provided their written informed consent to participate in this study.

## Author Contributions

Designed the study and writing of manuscript: JHS, MBH, and SRO. Acquisition of samples and registry data: OBP, HH, CE, HU, JD, LWT, MAHL, and ES. Collection of autoantibody data: JHS. Statistical analysis: JHS, OBP, HH, CE, HU, MBH, and SRO. All authors contributed to the article and approved the submitted version.

## Funding

The present study was funded by the Copenhagen University Hospital Research Fund (Project E-22461-03). DBDS has been funded by the Danish Regions and Bio- and Genome Bank Denmark.

## Conflict of Interest

The authors declare that the research was conducted in the absence of any commercial or financial relationships that could be construed as a potential conflict of interest.

## Publisher's Note

All claims expressed in this article are solely those of the authors and do not necessarily represent those of their affiliated organizations, or those of the publisher, the editors and the reviewers. Any product that may be evaluated in this article, or claim that may be made by its manufacturer, is not guaranteed or endorsed by the publisher.

## Supplementary Material

Supplementary Figure S1 IL-6 c-aAb vs IL-6 cytokine. IL-6 c-aAb MFI-values plotted against measured IL-6 concentration in plasma samples from the sub-study and grouped as either c-aAb low-titer (MFI < negative control +4SD) or c-aAb high-titer (MFI > 99th percentile) according to the original cohort screening.

Supplementary Table S1 IL-6 c-aAb as predictors of low platelet count or high mean platelet colume.

## Abbreviations

BM: Bone marrow
BMI: Body mass index
C-aAb: Cytokine-specific auto-antibodies
CI: Confidence interval
CRP: C-reactive protein
DBDS: The Danish Blood Donor Study
GM-CSF: Granulocyte macrophage colony-stimulating factor
IL: Interleukin
IFN: Interferon
MFI: Median fluorescence intensity
MPV: Mean Platelet Volume
OC: Oral contraceptives
OR: Odds ratio
SD: Standard deviation
TNF: Tumor necrosis factor
TPO: Thrombopoietin.
