# Supplementary figures and images for: IL-6 Autoantibodies Predict Lower Platelet Counts and Altered Plasma Cytokine Profiles in Healthy Blood Donors: Results From the Danish Blood Donor Study
Source: Front Med (Lausanne). 2022 Jun 24;9:914262. doi: 10.3389/fmed.2022.914262 (PMC9263719; doi:10.3389/fmed.2022.914262)

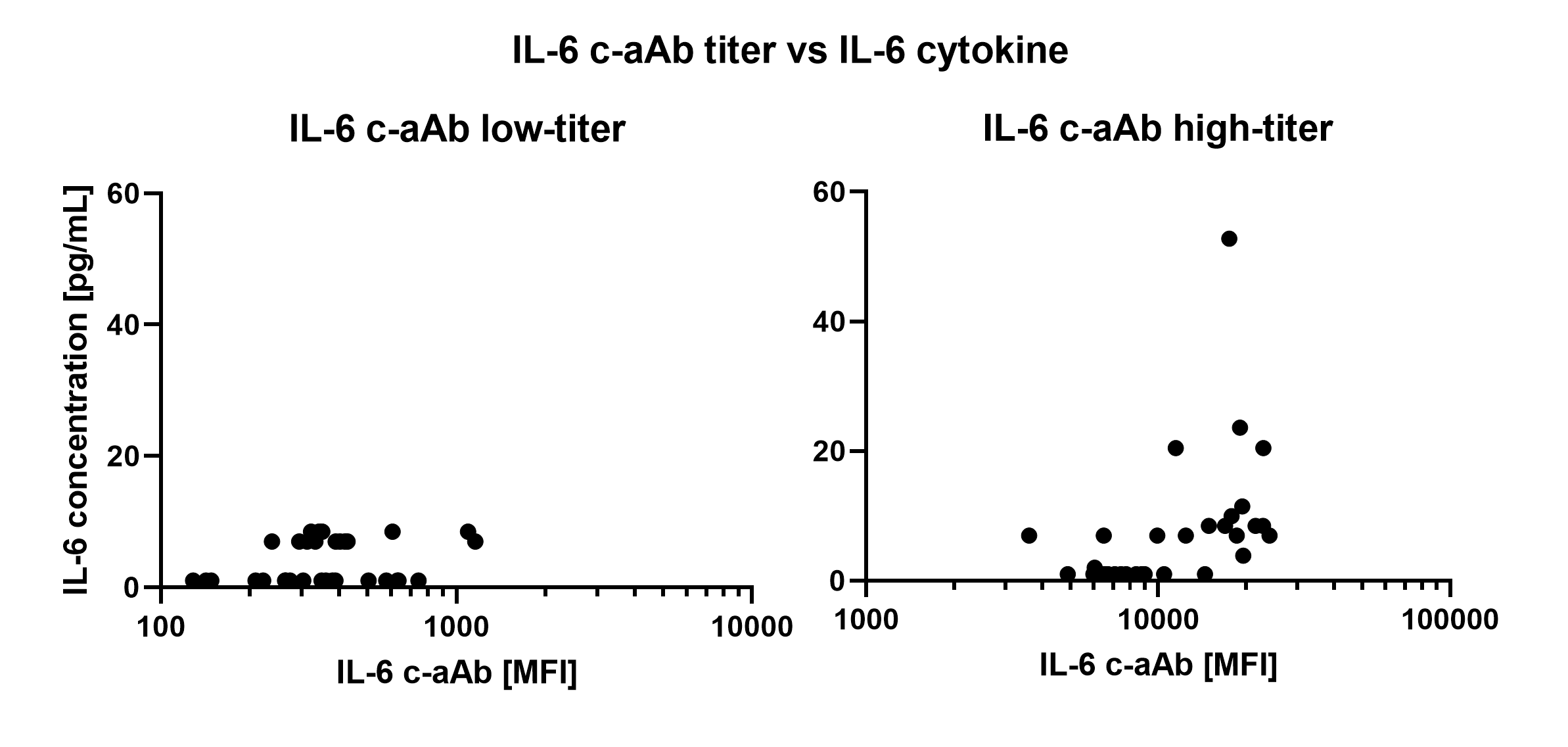

Supplement: Supplementary Figure S1 — IL-6 c-aAb vs IL-6 cytokine. IL-6 c-aAb MFI-values plotted against measured IL-6 concentration in plasma samples from the sub-study and grouped as either c-aAb low-titer (MFI < negative control +4SD) or c-aAb high-titer (MFI > 99th percentile) according to the original cohort screening. [file Image_1.TIF]
