# Supplementary material for: IL-6 Autoantibodies Predict Lower Platelet Counts and Altered Plasma Cytokine Profiles in Healthy Blood Donors: Results From the Danish Blood Donor Study
Source: Front Med (Lausanne). 2022 Jun 24;9:914262. doi: 10.3389/fmed.2022.914262 (PMC9263719; doi:10.3389/fmed.2022.914262)
Supplement: Supplementary Table S1 — IL-6 c-aAb as predictors of low platelet count or high mean platelet colume. [file Table_1.pdf]

**Table S1: IL-6 c-aAb as predictors of low platelet count or high mean platelet volume**						
**Sex**	**Platelet measurement**	**C-aAb titer**	**n**[a]	**Univariate Odds Ratio (95%CI)**	**Multivariate Odds Ratio (95%CI)**[b]	***p* value** [c]
Men	Low Platelet count[d]	Intermediary[f]	1,922	1.34 (0.96 – 1.90)	1.36 (0.95 – 1.95)	0.090
		High[g]		3.19 (1.14 – 8.92)	3.37 (1.19 – 9.62)	0.022
	High MPV[e]	Intermediary[f]	1,920	1.24 (0.91 – 1.72)	1.26 (0.90 – 1.71)	0.174
		High[g]		4.07 (1.62 – 10.22)	4.53 (1.76 – 11.61)	0.002
Women	Low Platelet count[d]	Intermediary[f]	1,643	0.82 (0.57 - 1.16)	0.78 (0.54 – 1.12)	0.184
		High[g]		N/A	N/A	N/A
	High MPV[e]	Intermediary[f]	1,640	0.94 (0.68 – 1.30)	0.93 (0.67-1.30)	0.697
		High[g]		0.46 (0.06 – 3.50)	0.44 (0.05 – 3.48)	0.444

a) Number of participants in adjusted logistic regressions

b) Adjusted for age, smoking, BMI, 1-year donation history, 1-year history of antimicrobial prescriptions, comorbidity, oral contraceptives and distance between c-aAb and closest platelet measurement. Whether an individual had low platelet count/ high MPV or not at the first platelet measurement within two years of c-aAb measurement was used as the dependent variable.

c) *P* values shown correspond to the adjusted regressions.

d) Low platelet count was defined as being below the 10th percentile of platelet count for the respective sex

e) High MPV was defined as being above the 90th percentile of platelet count for the respective sex

f) Intermediary c-aAb titer defined as MFI values above negative control +4 SD, but below the 99th percentile of MFI. Compared to c-aAb low individuals, with MFI< negative control +4 SD

g) High c-aAb titers defined as MFI values above the 99th percentile of MFI. Compared to c-aAb low individuals, with MFI< negative control +4 SD
